# A high-affinity antibody against the CSP N-terminal domain lacks *Plasmodium falciparum* inhibitory activity

**DOI:** 10.1084/jem.20200061

**Published:** 2020-08-13

**Authors:** Elaine Thai, Giulia Costa, Anna Weyrich, Rajagopal Murugan, David Oyen, Yevel Flores-Garcia, Katherine Prieto, Alexandre Bosch, Angelo Valleriani, Nicholas C. Wu, Tossapol Pholcharee, Stephen W. Scally, Ian A. Wilson, Hedda Wardemann, Jean-Philippe Julien, Elena A. Levashina

**Affiliations:** 1 Program in Molecular Medicine, The Hospital for Sick Children Research Institute, Toronto, Ontario, Canada; 2 Department of Biochemistry, University of Toronto, Toronto, Ontario, Canada; 3 Vector Biology Unit, Max Planck Institute for Infection Biology, Berlin, Germany; 4 B Cell Immunology, German Cancer Research Institute, Heidelberg, Germany; 5 Department of Integrative Structural and Computational Biology, The Scripps Research Institute, La Jolla, CA; 6 Department of Molecular Microbiology and Immunology, Malaria Research Institute, Johns Hopkins Bloomberg School of Public Health, Baltimore, MD; 7 Department of Theory and Biosystems, Max Planck Institute of Colloids and Interfaces, Potsdam, Germany; 8 The Skaggs Institute for Chemical Biology, The Scripps Research Institute, La Jolla, CA; 9 Department of Immunology, University of Toronto, Toronto, Ontario, Canada

## Abstract

Malaria is a global health concern, and research efforts are ongoing to develop a superior vaccine to RTS,S/AS01. To guide immunogen design, we seek a comprehensive understanding of the protective humoral response against *Plasmodium falciparum* (Pf) circumsporozoite protein (PfCSP). In contrast to the well-studied responses to the repeat region and the C-terminus, the antibody response against the N-terminal domain of PfCSP (N-CSP) remains obscure. Here, we characterized the molecular recognition and functional efficacy of the N-CSP–specific monoclonal antibody 5D5. The crystal structure at 1.85-Å resolution revealed that 5D5 binds an α-helical epitope in N-CSP with high affinity through extensive shape and charge complementarity and the unusual utilization of an antibody N-linked glycan. Nevertheless, functional studies indicated low 5D5 binding to live Pf sporozoites and lack of sporozoite inhibition in vitro and in vivo. Overall, our data do not support the inclusion of the 5D5 N-CSP epitope into the next generation of CSP-based vaccines.

## Teaser

Molecular characterization of inhibitory and non-inhibitory antibodies contributes to vaccine design. Here, we show that the high-affinity mAb 5D5 against the PfCSP N-terminus of sporozoites does not inhibit Pf invasion. This information is critical for the prioritization of functional sites of vulnerability to include in next-generation malaria immunogen designs.

## Introduction

Malaria is a vector-borne disease of global importance. In 2018, an estimated 228 million cases were reported, resulting in 405,000 deaths (World Health Organization, 2019). The majority of deaths are caused by *Plasmodium falciparum* (Pf), making this parasite a central focus of research efforts for the development of effective therapeutic interventions. Anti-infection vaccines target the sporozoite stage of the Pf life cycle as parasites are transmitted to the human host by infected female *Anopheles* mosquitoes during a blood meal. It was established four decades ago that mAbs targeting the sporozoite surface circumsporozoite protein (CSP) are capable of neutralizing *Plasmodium* infection (Potocnjak et al., 1980; Yoshida et al., 1980, 1981; Cochrane et al., 1982). This past year, the current leading anti-infection CSP-based vaccine against Pf malaria, RTS,S/AS01, began pilot implementation in Ghana, Malawi, and Kenya. Notwithstanding, RTS,S/AS01 was shown to only provide rapidly waning protection in 50% of children; thus, intense research efforts are underway toward designing a more efficacious and durable anti-CSP vaccine (RTS,S Clinical Trials Partnership, 2015; Julien and Wardemann, 2019).

**Figure 1.**
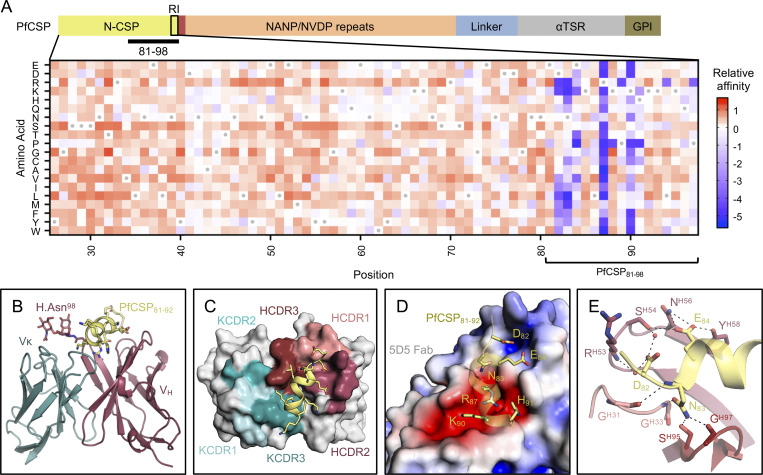
**Molecular delineation of the mAb 5D5 epitope in PfCSP.** **(A)** Top: Schematic depicting the protein domain organization of PfCSP, shown with the approximate location of RI indicated by the black box and the junctional epitope represented by a dark red band. An approximate representation of PfCSP_81–98_ is illustrated by the black bar (not shown to scale). Bottom: Heatmap of mAb 5D5 binding affinity for N-CSP single-point mutant library. N-CSP residues included in PfCSP_81–98_ are indicated by the bracket at the bottom. The relative binding affinity is indicated by a diverging color scale from red to blue, where red indicates a similar affinity while blue indicates decreased affinity. The x axis denotes the N-CSP residue position, and the y axis specifies the introduced single-point mutations. Residues corresponding to the WT sequence are indicated by the gray dots. **(B)** Crystal structure showing the 5D5 Fab variable regions (heavy chain shown in red and κ light chain shown in blue) bound to PfCSP N-terminal residues 81–92 (yellow), which are recognized in an α-helical conformation. The N-linked glycan on H.Asn98 of 5D5 Fab is represented as sticks. **(C)** mAb 5D5 CDRs contacting PfCSP. HCDRs 1, 2, and 3 (salmon, raspberry, and firebrick red, respectively) and KCDRs 1 and 3 (light teal and deep teal, respectively) contribute to 5D5 Fab recognition, whereas KCDR2 (teal) does not. **(D)** Electrostatic surface potential of mAb 5D5 bound to PfCSP_81–92_. mAb 5D5 displays extensive shape and charge complementarity to PfCSP. Electrostatic calculations were performed using Adaptive Poisson-Boltzmann Solver (APBS; Baker et al., 2001) and rendered in PyMOL (The PyMOL Molecular Graphics System, Version 2.0; Schrödinger, LLC); scale: −5 kT/e (red) to +5 kT/e (blue). **(E)** H-bonds (shown as black dashed lines) formed between mAb 5D5 HCDR residues and negatively charged PfCSP residues. Water molecules are shown as red spheres. αTSR, α-thrombospondin type 1 repeat. GPI, glycosylphosphatidylinositol. kT/e, unit of electrostatic potential.

A molecular understanding of how the most potent mAbs recognize sites of vulnerability on the parasite can guide next-generation vaccine design. Pf circumsporozoite protein (PfCSP) is composed of an N-terminal domain (N-CSP), a central repeat region comprising NANP motifs of varied numbers that are interspersed with related NVDP motifs, and a C-terminal domain (C-CSP) that comprises a linker region preceding an α-thrombospondin type-1 repeat domain (Fig. 1 A). PfCSP is linked to the parasite membrane through a glycosylphosphatidylinositol anchor site. Numerous studies have shown that mAbs specific for the NANP repeat region and the junction immediately following N-CSP, which contains NANP motifs, NVDP motifs, and the only copy of an NPDP motif, can mediate protection in animal models (Potocnjak et al., 1980; Yoshida et al., 1980; Foquet et al., 2014; Oyen et al., 2017; Triller et al., 2017; Kisalu et al., 2018; Tan et al., 2018; Imkeller et al., 2018; Murugan et al., 2020). The few mAbs to C-CSP that have been described were ineffective, probably due to low accessibility of this domain on the sporozoite surface (Scally et al., 2018).

In contrast, the functional relevance of N-CSP mAbs remains elusive. To date, no human mAb specific for this domain and only a handful of murine mAbs from immunization studies with recombinant Pf N-CSP have been reported (Espinosa et al., 2015; Herrera et al., 2015). These mAbs recognized N-CSP epitopes adjacent to Region I (RI; Fig. 1 A), a site with high conservation across *Plasmodium* species, suggesting that RI may be a good target for cross-species vaccine development (Dame et al., 1984; Espinosa et al., 2015). Additionally, proteolytic cleavage of RI was linked to efficient sporozoite invasion of host hepatocytes (Espinosa et al., 2015; Coppi et al., 2005). Based on these observations, it has been proposed that adding N-CSP, including the RI motif, into a PfCSP subunit vaccine may improve protective efficacy compared to the current leading vaccine RTS,S/AS01, which lacks this domain. However, passive transfer of the most potent N-CSP–specific murine mAb 5D5, whose epitope lies immediately upstream of RI, protected mice from infection in only one of the two tested transgenic rodent *Plasmodium berghei* (Pb) liver burden models that expressed a chimeric PbCSP with the Pf N-CSP domain (Espinosa et al., 2015), and its impact on Pf has not been determined. Thus, crucial information on how mAb 5D5 binds and inhibits Pf sporozoites is still missing.

To gain a molecular understanding of how the mAb 5D5 recognizes PfCSP and inhibits Pf sporozoite infectivity, we solved the crystal structure of the 5D5 Fab in complex with a peptide derived from N-CSP and conducted in-depth binding and functional experiments with Pf sporozoites. We specifically quantified reactivity of mAb 5D5 to single live Pf sporozoites isolated from the midgut and salivary glands of mosquitoes using imaging flow cytometry and tested its inhibitory potency against Pf sporozoites by in vitro and in vivo assays. By providing a detailed molecular and functional understanding of mAb 5D5 recognition of its epitope, we report that this anti–N-CSP antibody inefficiently binds to live Pf sporozoites and lacks inhibitory activity.

## Results and discussion

### mAb 5D5 binds an α-helical motif in N-CSP

**Figure S1.**
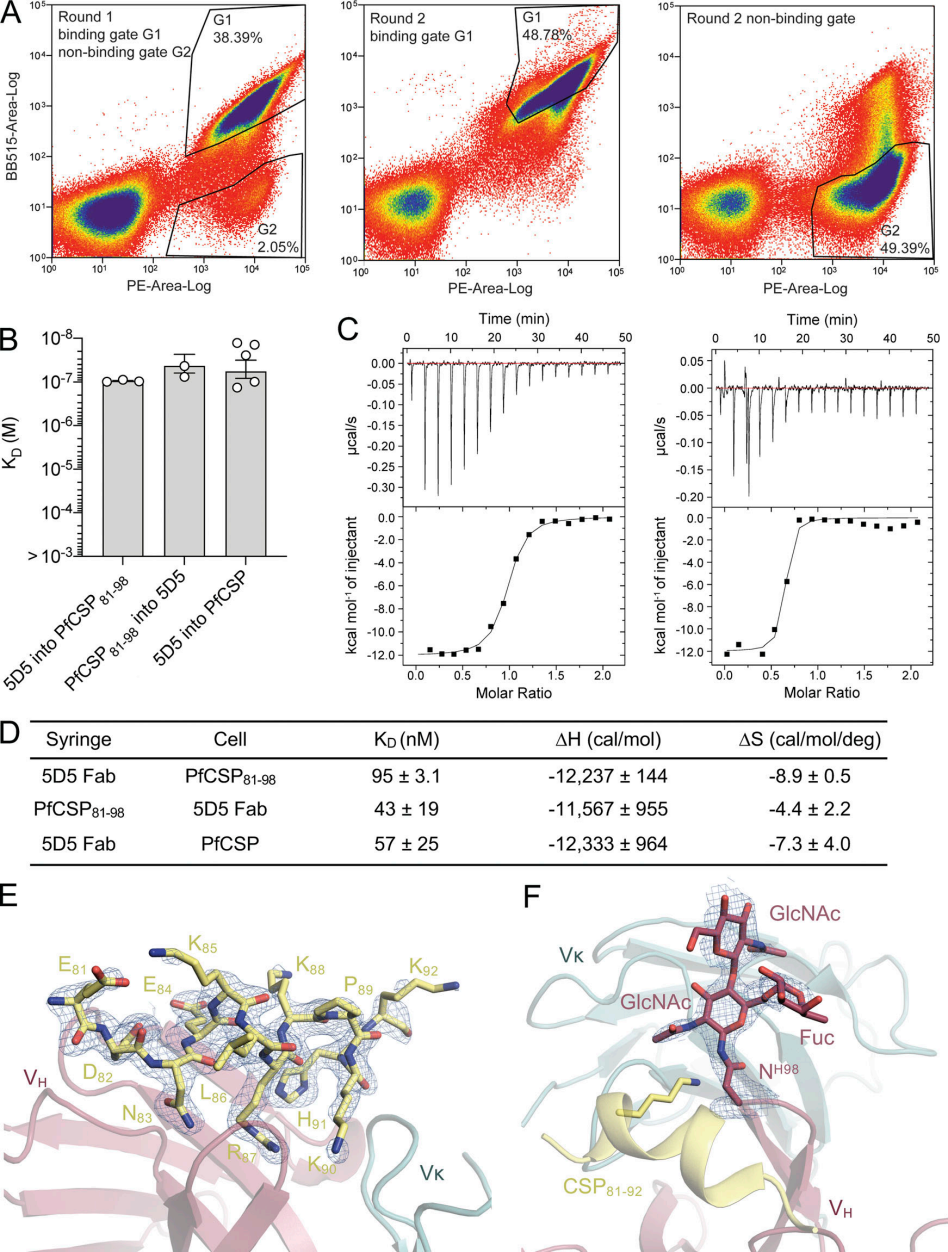
**Experimental details of FACS of 5D5 IgG yeast display epitope mapping library, comparison of 5D5 Fab binding to PfCSP_81_**_–_**_98_ and PfCSP, and electron density observed from crystallographic studies.** **(A)** Results from cell sorting (FACS) are shown. Left: Round 1 sorting with 5D5 IgG binding (G1) and nonbinding (G2) gates. Middle: Round 2 sorting with 5D5 IgG binding gate. Right: Round 2 sorting with 5D5 IgG nonbinding gate. Percentages of cells are shown within the drawn gates. **(B)** Affinities of 5D5 Fab for PfCSP_81–98_ and PfCSP as measured by isothermal titration calorimetry (ITC). Symbols represent independent measurements, and error bars represent SEM. At least three independent measurements were made for each binding interaction. **(C)** Representative plots of 5D5 Fab titrating into PfCSP_81–98_ (left) and PfCSP (right) at 25°C. Above: Raw data of PfCSP_81–98_ (0.01 mM) or PfCSP (0.005 mM) in the sample cell titrated with 5D5 Fab (0.1 mM and 0.05 mM, respectively). Below: Plot and trendline of heat of injectant corresponding to the raw data. Analysis was performed using the Malvern MicroCal ITC Analysis software. Data are representative of three independent measurements for PfCSP_81–98_ and five independent measurements for PfCSP. **(D)** Mean K_D_ values and thermodynamic parameters of 5D5 Fab binding to PfCSP_81–98_ and PfCSP as measured by ITC for at least three independent experimental replicates. SEM is reported. **(E and F)** Composite omit map electron density contoured at 1 σ (blue mesh) around the N-CSP peptide (E) and the 5D5 N-linked glycan at position H.Asn98 of the HCDR3 (F). H, enthalpy. S, entropy.

To understand the molecular basis for mAb 5D5 recognition of PfCSP, we solved the crystal structure of the 5D5 Fab in complex with PfCSP_81–98_ to 1.85-Å resolution (Table S1). We specifically selected PfCSP residues 81–98 for our studies to ensure inclusion of the mAb 5D5 epitope, identified as Pf N-CSP residues 82–91 by yeast display epitope mapping (Fig. 1 A and Fig. S1 A) in agreement with previous reports (Espinosa et al., 2015), as well as conserved RI residues KLKQP in positions 93–97. Isothermal titration calorimetry further confirmed that the PfCSP_81–98_ peptide comprised the majority of the 5D5 epitope, as 5D5 Fab bound equally well to PfCSP_81–98_ and full-length PfCSP (Fig. S1, B–D). Consistent with these experiments, we observed strong electron density for N-CSP residues 81–92 (EDNEKLRKPKHK) in the crystal structure (Fig. S1 E). PfCSP residues 83–91 formed an α-helix when bound by 5D5 Fab (Fig. 1 B), in line with secondary structure predictions based on the primary sequence (Drozdetskiy et al., 2015). Importantly, while structures of a variety of polypeptides derived from PfCSP (the junctional region following N-CSP, the NANP repeat region, and the C-terminal α-thrombospondin type 1 repeat domain) have been solved in complex with a broad range of antibodies (Oyen et al., 2017; Imkeller et al., 2018; Scally et al., 2018; Tan et al., 2018; Kisalu et al., 2018; Julien and Wardemann, 2019; Murugan et al., 2020; Pholcharee et al., 2020; Oyen et al., 2020), our crystal structure of the 5D5 Fab in complex with PfCSP_81–98_ provides the first insight into the subdomain architecture of Pf N-CSP. However, further studies are needed to elucidate the conformation of residues comprising RI, which were disordered and unresolved in our crystal structure, as well as the overall structure of Pf N-CSP.

**Figure 2.**
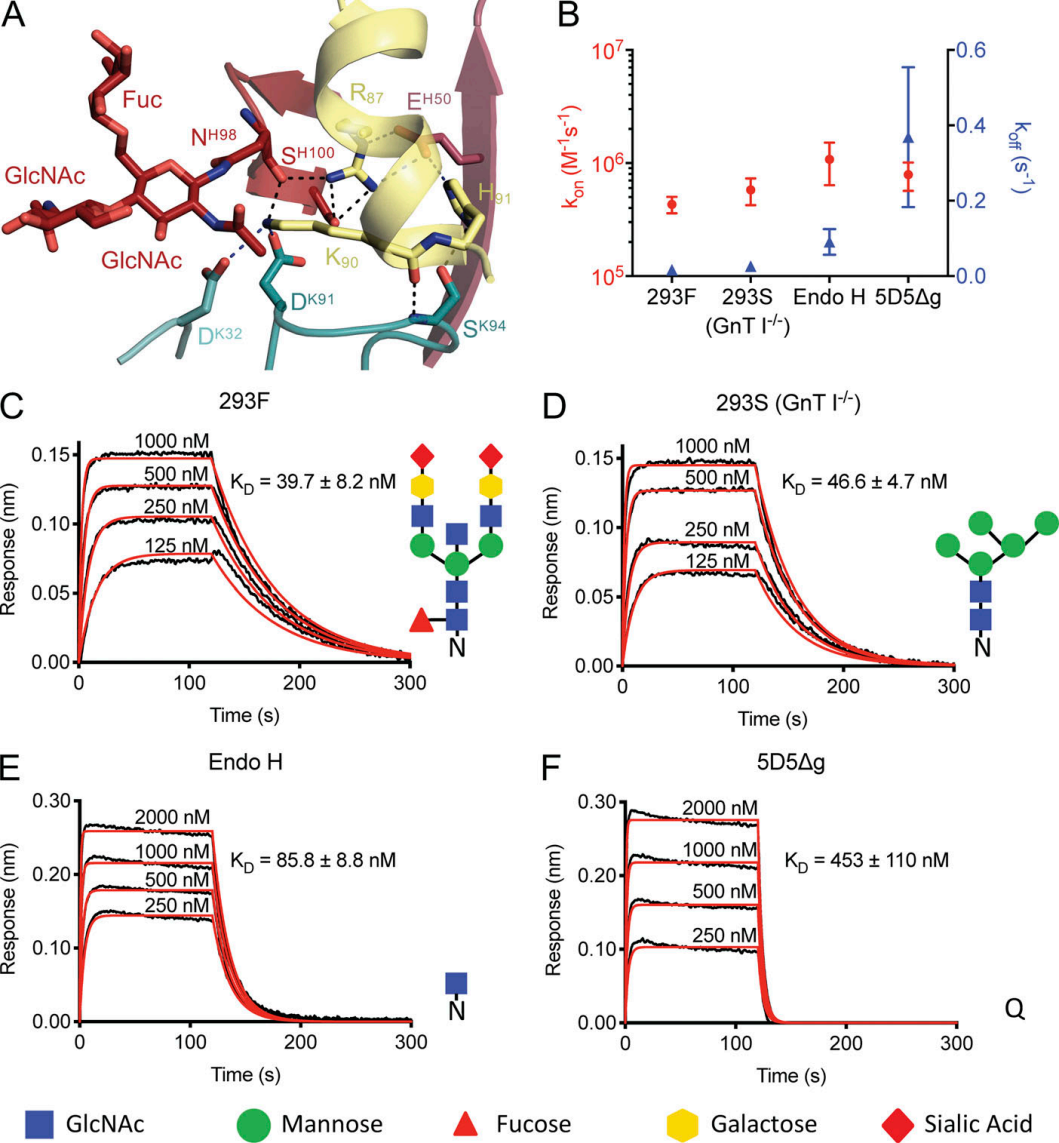
**5D5 paratope glycosylation mediates high-affinity binding.** **(A)** Interactions formed by mAb 5D5 H.Asn98-linked GlcNAc moiety and surrounding CDR residues with PfCSP. H-bonds are shown as black dashed lines, and salt bridges are shown as blue dashed lines. **(B–F)** Binding kinetics of twofold dilutions of 293F (C), 293S (GnT I^−/−^; D), Endo H (E), and 5D5Δg (F) Fab glycoform variants to full-length PfCSP. **(B)** Mean on- and off-rates (k_on_ and k_off_, respectively) of the 5D5 Fab glycoform variants binding to full-length PfCSP are plotted on the left and right y axes, respectively, for three independent experimental replicates. Mean k_on_ rates are shown as red circles, and mean k_off_ rates as blue triangles. Error bars represent one SD from the mean. **(C–F)** Representative sensorgrams are shown in black and 1:1 model best fits in red. Mean K_D_ values are as listed. K_D_ values and k_on_ and k_off_ rates were determined by FortéBio’s Data Analysis software 9.0. Standard error values are reported as the SD. Data are representative of three independent measurements. Corresponding glycan structures are shown using symbols adhering to the Symbol Nomenclature for Glycans (Varki et al., 2015).

mAb 5D5 contacts PfCSP with all complementarity-determining regions (CDRs) except κ light chain CDR 2 (KCDR2; Fig. 1 C). The heavy chain CDRs (HCDRs) form the majority of interactions, with 498 Å^2^ buried surface area (BSA) compared to 160 Å^2^ BSA for the κ light chain. Furthermore, the mAb 5D5 CDRs possess extensive shape and electrostatic complementarity to this highly charged N-CSP epitope (Fig. 1 D). An electropositive pocket formed by HCDR2 contacts PfCSP residues D82 and E84 via H-bonds with Ser54^Oγ^, Asn56^Nδ2^, and Tyr58^OH^ and water-mediated H-bonds with Arg53^N^ and Ser54^Oγ^ (Fig. 1 E). Additionally, an electronegative pocket formed by HCDR2, HCDR3, KCDR1, and KCDR3 contacts PfCSP electropositive residues R87, K90, and H91 via several H-bonds and salt bridges (Fig. 2 A). The significance of this shape and charge complementarity for high-affinity binding was observed in our yeast display experiments, as mutations maintaining both side chain length and electrostatic properties, such as K90R, were more likely to sustain high-affinity binding than those that did not (K90E and K90D; Fig. 1 A).

**Figure S2.**
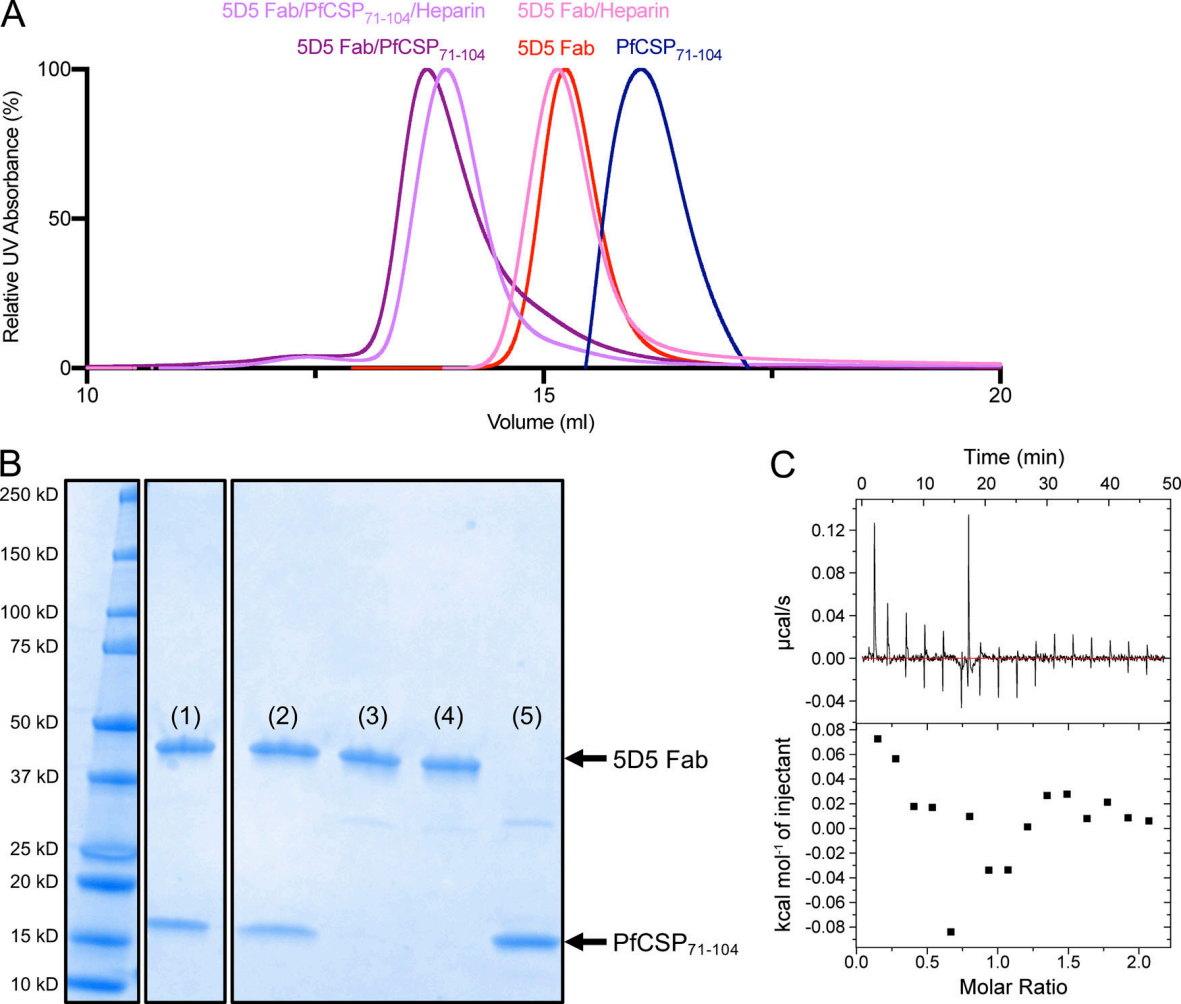
**Investigation of heparin binding to Pf N-CSP.** **(A)** Size-exclusion chromatography examination of 5D5 Fab in complex with N-CSP coincubated with heparin. Chromatograms of 5D5 Fab (red), 5D5 Fab incubated with 100-fold molar excess of heparin sodium salt (pink), PfCSP_71–104_ (blue), 5D5 Fab in complex with PfCSP_71-104_ (purple), and 5D5 Fab in complex with PfCSP_71–104_ coincubated with 100-fold molar excess of heparin sodium salt (lilac) are shown. Data are representative of three different experiments probing these interactions. **(B)** SDS-PAGE analysis of the following peaks from size-exclusion chromatography experiments, examined in nonreducing conditions: (1) 5D5 Fab/PfCSP_71–104_, (2) 5D5 Fab/PfCSP_71–104_/heparin, (3) 5D5 Fab/heparin, (4) 5D5 Fab, and (5) PfCSP_71–104_. **(C)** Representative isothermal titration calorimetry data (top) and resulting plot of heat of injectant (bottom) of heparin sodium salt (2,000 µM) titrated into PfCSP_81–98_ (200 µM) at 25°C. Analysis was performed using the Malvern MicroCal isothermal titration calorimetry Analysis software. Data are representative of five different experiments probing this interaction.

Consistent with the prediction from the primary mAb 5D5 sequence, we observed electron density for two GlcNAc and one α1-6Fuc moieties indicative of N-linked glycosylation at H.Asn98 of HCDR3 (Fig. 2 A and Fig. S1 F). Importantly, the first N-linked GlcNAc moiety contacts the aliphatic portion of K90 of PfCSP_81–98_, conferring 48 Å^2^ of BSA, while the other sugars did not interact with the peptide. In this way, the paratope glycan contributes to mAb 5D5 occlusion of PfCSP residue K90. Interestingly, K90 is one of four lysine residues (including K85, K88, and K92) directly upstream of RI that were previously proposed to be important for binding heparan sulfate proteoglycans on the surface of hepatocytes to initialize liver invasion (Zhao et al., 2016). Notably, the α-helical conformation adopted by N-CSP residues 83–91 upon mAb 5D5 binding positions the remaining three lysine residues, K85, K88, and K92, on the same exposed face of the helix (Fig. S1 E). However, because the putative binding interaction between Pf N-CSP and heparin is very weak (Fig. S2), we did not further investigate the role of mAb 5D5 in inhibiting PfCSP binding to heparan sulfate proteoglycans. Our molecular description of PfCSP recognition by mAb 5D5 demonstrates optimal antibody–antigen characteristics associated with high-affinity binding to a putative functional site on Pf sporozoites.

### mAb 5D5 paratope glycosylation is critical for high-affinity recognition of recombinant PfCSP

To determine whether the N-linked glycan on H.Asn98 affects mAb 5D5 binding, we generated four different forms of the mAb 5D5 glycan and measured their binding kinetics to full-length PfCSP using biolayer interferometry (BLI; Fig. 2, B–F). Specifically, we generated four 5D5 Fab variants with (1) a complex glycan, as in the crystal structure (by expression in HEK293F cells; 293F); (2) a high mannose glycan (by expression in HEK293S [GnT I^−/−^] cells; 293S [GnT I^−/−^]); (3) a single GlcNAc moiety (by expression in HEK293S [GnT I^−/−^] cells followed by endoglycosidase H treatment [Endo H]); or (4) an H.N98Q mutation removing the N-linked glycosylation site altogether (5D5Δg). The 293F-, 293S (GnT I^−/−^)–, and Endo H–treated 5D5 Fabs bound with high affinity to full-length PfCSP with a K_D_ of 39.7 ± 8.2 nM, 46.6 ± 4.7 nM, and 85.8 ± 8.8 nM, respectively (Fig. 2, C–E). In contrast, the 5D5Δg mutant Fab bound PfCSP with weaker affinity (K_D_ of 453 ± 110 nM) due to an 11-fold faster off-rate compared with HEK293F–expressed 5D5 Fab (Fig. 2, B and F), while the on-rates of all glycoform Fabs remained within the same order of magnitude. Together with the crystal structure, these results underline the importance of the H.Asn98-linked GlcNAc moiety for high-affinity mAb 5D5 binding to recombinant PfCSP and highlight a rare occurrence for such a post-translational modification to participate in the antibody interaction with antigen and improve the kinetics of antigen binding.

### mAb 5D5 does not efficiently bind or inhibit salivary gland sporozoites

**Figure S3.**
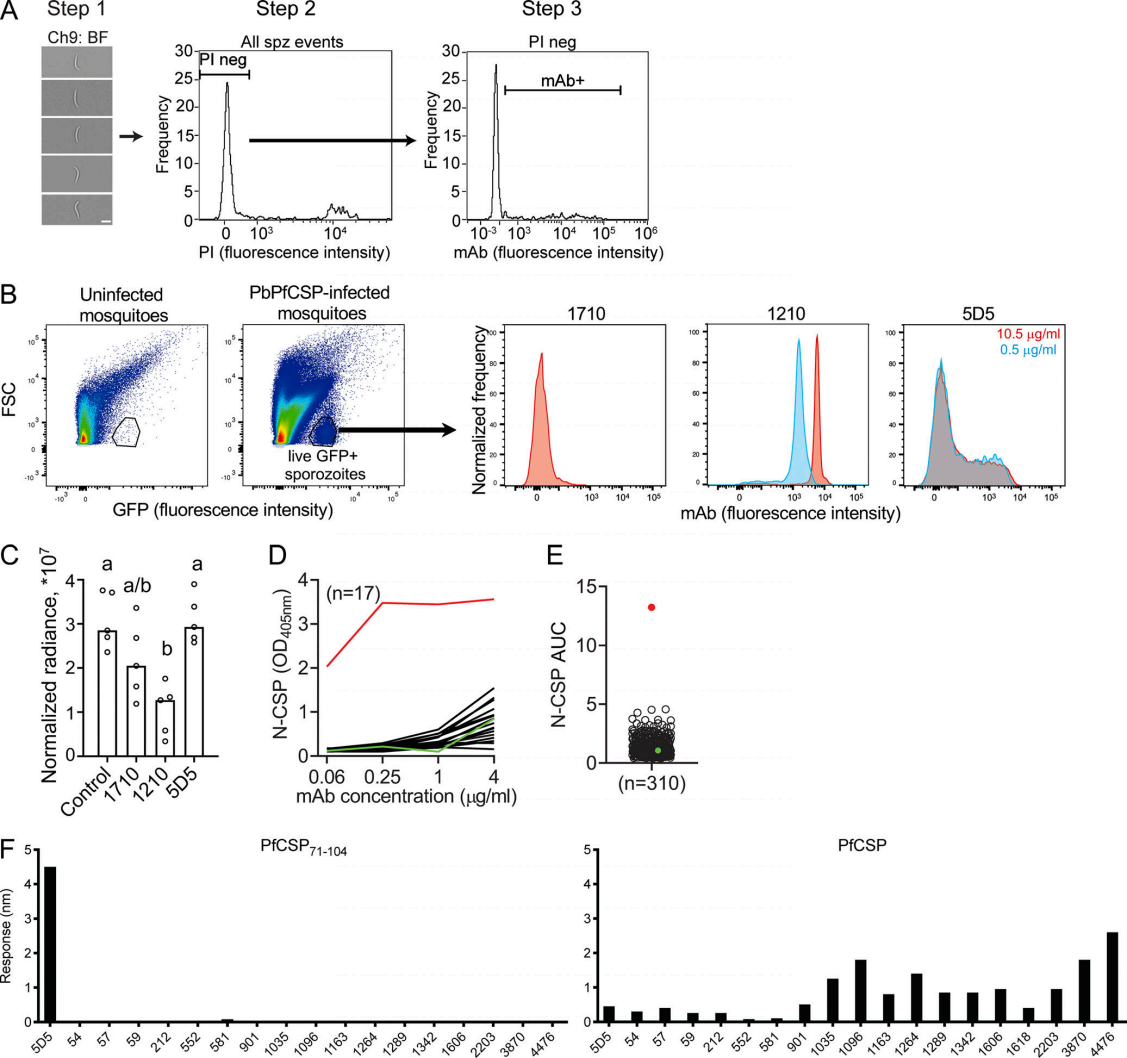
**Gating strategy for imaging flow cytometry quantification of mAb binding to live Pf and PbPfCSP sporozoites, passive immunization liver burden assay, and identification of human mAbs against N-CSP in the PfSPZ-CVac samples.** **(A)** The gating strategy for imaging flow cytometry of mAb binding to live Pf sporozoites included three steps. Step 1: single in-focus sporozoites were manually selected on brightfield (BF) images (scale bar: 5 µm). Step 2: live sporozoites were gated as the propidium iodide (PI)–negative population. Step 3: the proportion and MFI of mAb-bound sporozoites were quantified. **(B)** Effect of concentration (0.5 and 10.5 µg/ml) on mAb binding to live PbPfCSP sporozoites (Triller et al., 2017) by FACS analysis. Uninfected mosquito material was used as a gating control and live sporozoites were identified as the GFP-positive population (*n* = 1). **(C)** Passive immunization liver burden assay. Mice were intravenously injected with mAbs (100 µg/mouse) before intravenous injection of PbPfCSP sporozoites (2,000 sporozoites/mouse; Flores-Garcia et al., 2019). Control mice did not receive any injection before infection. Radiance levels were normalized by subtracting the background luminescence levels in uninfected mice (*n* = 5 mice per group, *n* = 1). Statistically significant differences between the groups are indicated by different letters (P < 0.05, Kruskal-Wallis unpaired test followed by Dunn's post hoc test). Serum concentration of the mAbs 1710 (44.8 ± 5.2 ng/µl), 1210 (48.4 ± 4.7 ng/µl), and 5D5 (11.9 ± 1.2 ng/µl) were measured by ELISA using samples collected 15 h after passive transfer. The values represent mean and SD of five mice per mAb group, each measured three independent times. Lower levels of mAb 5D5 compared with mAbs 1210 and 1710 were within the range of values typically observed for these experiments (Raghunandan et al., 2020; Murugan et al., 2020). **(D)** Binding of human mAbs isolated from memory B cells and plasmablasts (Murugan et al., 2018) to N-CSP as measured by ELISA at the indicated antibody concentrations (*n* = 17). Red and green lines indicate mAb 5D5 (positive control) and mAb mGO53 (negative control), respectively. **(E)** Area under the ELISA curve (AUC) calculated from D for mAbs (*n* = 310). Red and green dots indicate mAbs 5D5 and mGO53, respectively. **(D and E)** Data are representative of at least two independent measurements. **(F)** Binding of 17 human mAbs expressed as IgGs with an N-CSP AUC > 3 in at least one ELISA from E to PfCSP_71–104_ (left) and PfCSP (right) by BLI. 5D5 IgG was used as a positive control. Data are representative of two independent measurements. spz, sporozoite. FSC, forward scatter.

**Figure 3.**
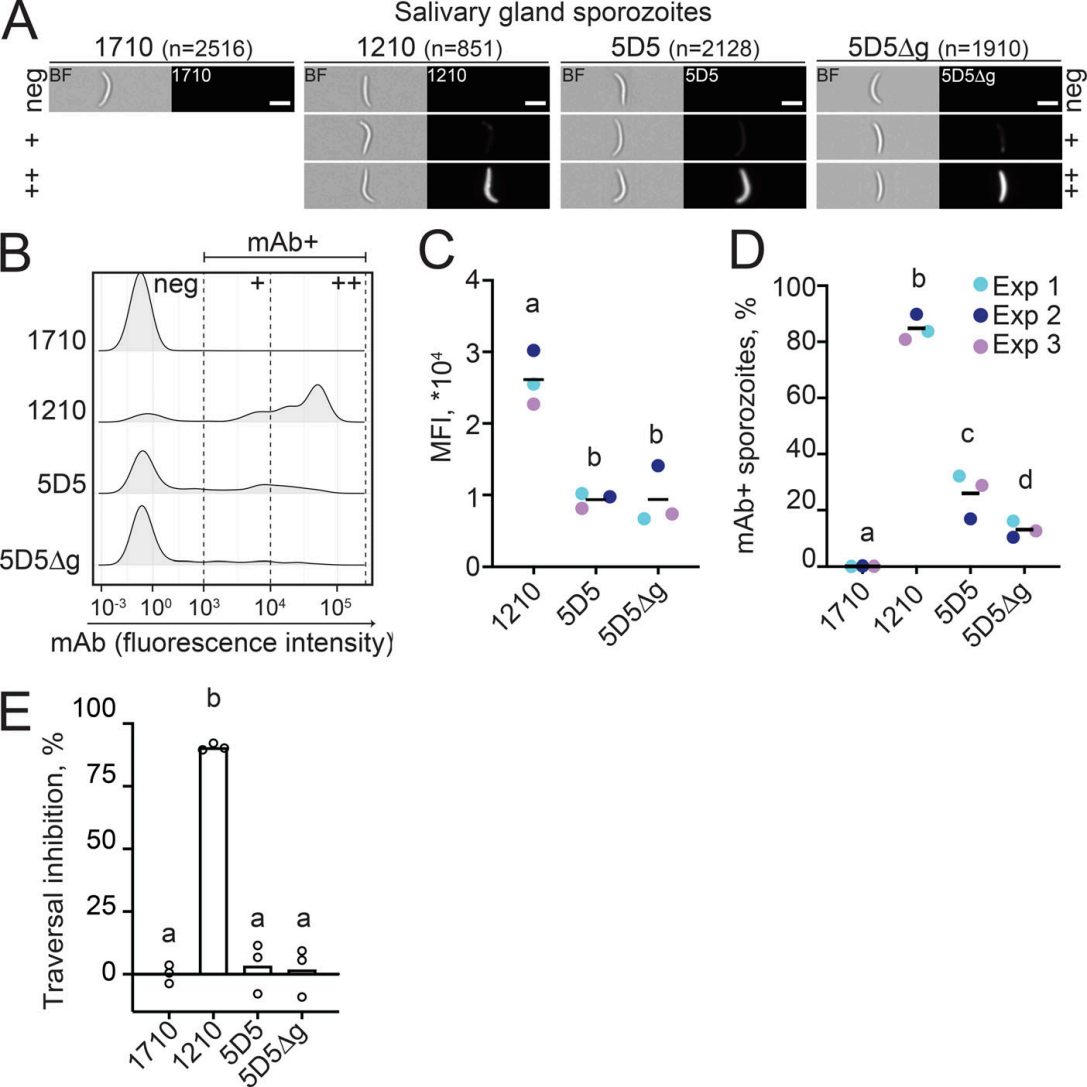
**mAb 5D5 binding and inhibition of mature salivary gland sporozoites.** **(A–D)** Imaging flow cytometry of live salivary gland sporozoites isolated from the mosquito thorax after incubation with human mAb 1710 or 1210 (negative and positive controls, respectively), or mAb 5D5 or 5D5Δg. **(A)** Representative images of sporozoites in brightfield (BF, left panels) and mAb-bound fluorescent sporozoites (right panels). Scale bars: 5 µm. Total number of sporozoites (*n*) analyzed per condition is indicated in parentheses (*n* = 3). **(B)** Comparative density plots of a representative experiment showing the fluorescence intensities of three arbitrarily designated groups of mAb-bound sporozoites (neg, negative; +, low intensity; ++, high intensity). **(C)** MFI of the mAb-positive sporozoites. **(D)** Quantification of mAb-positive sporozoites (%). **(C and D)** Colors show results of independent experiments (Exp). Horizontal black lines indicate means of three independent experiments. **(E)** Results of mAb inhibition of sporozoites in in vitro traversal assay tested at 100 µg/ml mAb concentration (*n* = 3). **(C–E)** Statistically significant differences (P < 0.05) between the groups are indicated by different letters (z-test [C and D]; paired Friedman test followed by Dunn’s post hoc test [E]).

The role of the N-linked glycan on H.Asn98 in the binding of 5D5 IgG to freshly isolated salivary gland Pf sporozoites was quantified by imaging flow cytometry. As these preparations contained both live and dead sporozoites, we focused our analyses on live sporozoites that were negative for propidium iodide staining (Fig. S3 A). As positive and negative controls, we used human mAbs targeting the PfCSP central repeat (1210; Imkeller et al., 2018) and C-CSP (1710; Scally et al., 2018), respectively. In line with previous reports, mAb 1710 failed to recognize mature Pf sporozoites isolated from mosquito salivary glands, whereas mAb 1210 strongly bound these sporozoites (Fig. 3, A–D). We detected a twofold decrease in mean fluorescence intensity (MFI) between mAbs 1210– and 5D5– or 5D5Δg–bound sporozoites (Fig. 3 C). The observed differences can be explained by the frequencies of the targeted epitopes on the sporozoite surface. Indeed, mAb 1210 likely binds repeated NANP motifs within the central region, whereas mAbs 5D5 and 5D5Δg can only react with a single N-CSP motif. In contrast to previous reports, we found that mAb 5D5 did not bind the majority of sporozoites (Fig. 3, B and D). This observed lack in sporozoite binding was not due to low antibody concentration, as even a 10-fold increase in mAb 5D5 concentration did not increase the proportion of mAb 5D5–bound sporozoites (Fig. S3 B). Mutation of the glycosylation site further decreased the proportion of 5D5Δg-bound sporozoites from 27% to 13% (Fig. 3 D). These results demonstrate the importance of mAb 5D5 paratope glycosylation for PfCSP binding on the sporozoite surface. However, they also reveal low levels of overall reactivity of this antibody to live salivary gland Pf sporozoites.

We next evaluated how the low sporozoite binding observed for mAb 5D5 translated into inhibitory potency against Pf sporozoites in a hepatocyte traversal assay. In line with the mAb binding efficiencies, only mAb 1210 completely blocked sporozoite traversal of hepatocytes, whereas mAb 5D5 was as inefficient at inhibiting traversal as negative control mAb 1710, regardless of the presence of the paratope glycan (Fig. 3 E). Independently, we failed to detect inhibitory activity from mAb 5D5 in in vivo passive immunization experiments using a Pb model system where mice were intravenously injected with transgenic Pb sporozoites expressing full-length PfCSP (PbPfCSP; Fig. S3 C). Altogether, we conclude that the low levels of mAb 5D5 binding to live sporozoites do not protect against parasite infection.

### mAb 5D5 does not inhibit in vivo sporozoite development in mosquitoes

**Figure 4.**
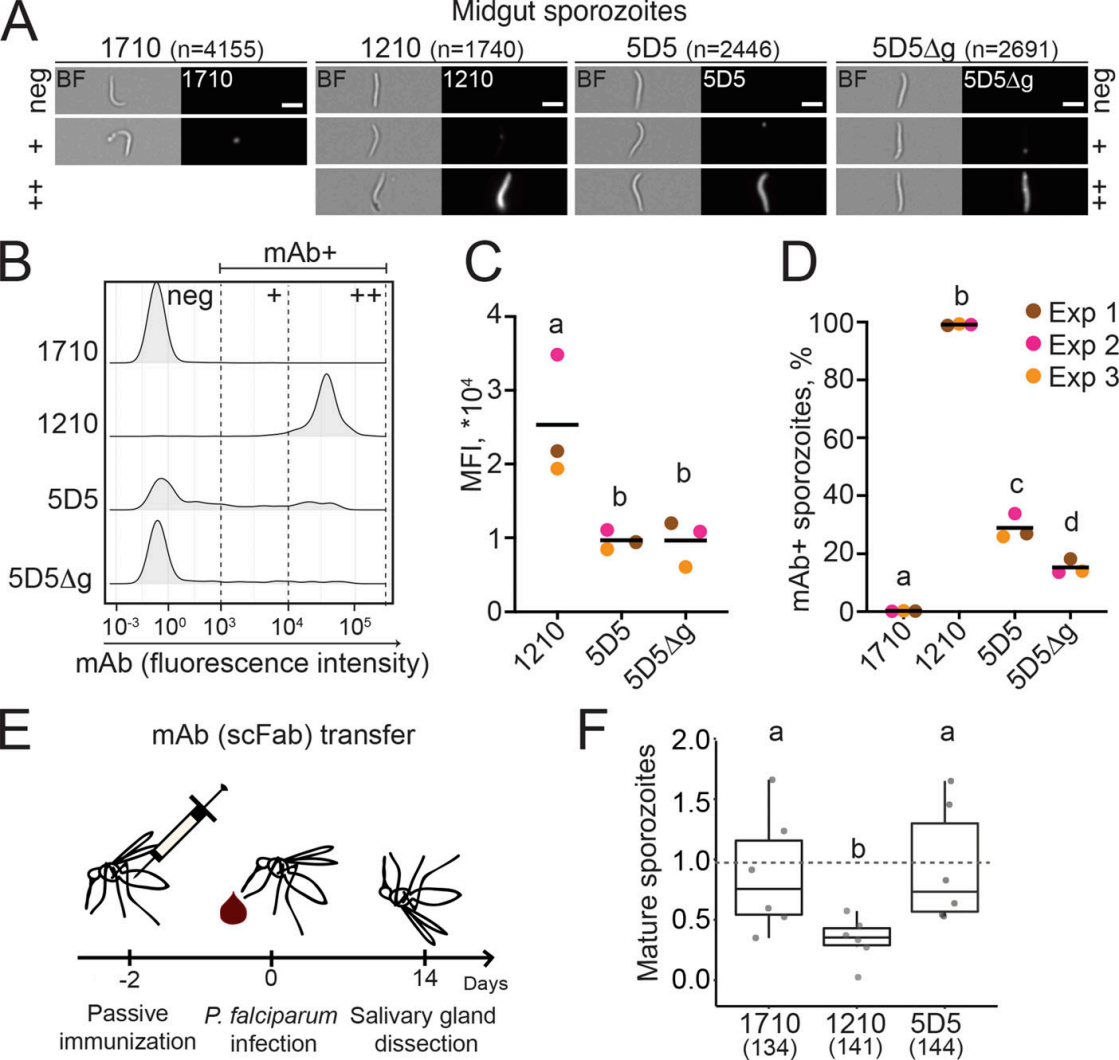
**mAb 5D5 binding to midgut sporozoites and inhibition of sporogonic development within mosquitoes.** **(A–D)** Imaging flow cytometry of live midgut sporozoites isolated from oocysts after incubation with human mAb 1710 or 1210 (negative and positive controls, respectively), or mAb 5D5 or 5D5Δg. **(A)** Representative images of sporozoites in brightfield (BF; left panels) and mAb-bound fluorescent sporozoites (right panels). Scale bars: 5 µm. Total number of sporozoites (*n*) analyzed per condition is indicated in parentheses (*n* = 3). **(B)** Comparative density plots of a representative experiment showing the fluorescence intensities of three arbitrarily designated groups of mAb-bound sporozoites (neg, negative; +, low intensity; ++, high intensity). **(C)** MFIs of the mAb-positive sporozoites. **(D)** Quantification of mAb-positive sporozoites (%). **(C and D)** Colors show results of independent experiments (Exp). Horizontal black lines indicate means of three independent experiments. **(E)** Schematic representation of passive scFab transfer by mosquito injection. **(F)** Results of scFab transfer experiments expressed relative to control PBS-injected mosquitoes (*n* = 6; total mosquito numbers analyzed are indicated below in parentheses). The box plots show the upper and lower quantiles and the median of the distribution; whiskers indicate min and max range. Each dot represents sporozoite loads per mosquito in one experiment normalized to control mosquitoes. Statistically significant differences (P < 0.05) between the groups are indicated by different letters (z-test [C and D]; MLE [F]).

As CSP is essential for sporozoite development in mosquitoes (Ménard et al., 1997), we extended our examination of antibody binding and function to immature Pf sporozoites isolated from oocysts in the mosquito midgut (Fig. 4). Similar to our observations with mature sporozoites, mAb 5D5 exhibited low binding efficiency to immature sporozoites, as measured by MFI and percentage of mAb-positive sporozoites determined using imaging flow cytometry (Fig. 4, A–D). Also consistent with our findings with salivary gland sporozoites, paratope glycosylation increased the proportion of mAb 5D5–bound midgut sporozoites by twofold (Fig. 4 D). Overall, our imaging analyses of mAb 5D5 binding to live sporozoites showed no difference between these two maturation stages. Thus, more detailed investigation of PfCSP organization on the sporozoite surface will be required to determine whether the minority population of mAb 5D5–bound sporozoites exhibit an alternative PfCSP conformation, incomplete cleavage, or other unusual attributes.

To evaluate the inhibitory activity of mAb 5D5 against Pf sporozoites in their natural environment in vivo, we developed a passive mAb transfer assay for mosquitoes that examined sporozoite maturation and salivary gland invasion. Mosquitoes were injected with recombinant single-chain Fabs (scFabs) 2 d before Pf infection, and sporozoite loads in dissected salivary glands were quantified 2 wk later (Fig. 4 E). Injection of scFab1210 significantly reduced the number of mature sporozoites in the salivary glands. In contrast, transfer of scFab1710 or scFab5D5 did not affect sporozoite development and invasion (Fig. 4 F). Therefore, these results reveal that mAb 5D5 fails to efficiently recognize its epitope on the surface of Pf parasites and lacks inhibitory potency against sporozoites in both the vector and the host.

### Concluding remarks

In this report, we demonstrate that despite high-affinity binding to recombinant PfCSP, mAb 5D5 does not recognize a majority of live Pf sporozoites, indicating that its epitope is not readily accessible or present on the sporozoite surface. The lack of potent human N-CSP–specific mAbs in multiple screens based on recombinant PfCSP baits or unbiased antigen-agnostic approaches (Fig. S3, D–F; Triller et al., 2017; Murugan et al., 2018; Tan et al., 2018; Kisalu et al., 2018; Julien and Wardemann, 2019) is in line with these observations. Consequently, as shown in the current study, mAb 5D5 is unable to block sporozoite development in the mosquito or inhibit sporozoite hepatocyte invasion in vitro and in vivo. Overall, to date, there is little evidence to support N-CSP as a source of potent or protective epitopes to block Pf infection. In the absence of potent anti–N-CSP mAbs, the repeating motifs in the central domain and N-terminal junction remain the most promising PfCSP regions to elicit protective mAbs and should be prioritized for anti-infective vaccine design.

## Materials and methods

### Mutant N-CSP yeast display library construction and transformation

Epitope mapping using phage display was adapted from a previously published method (Van Blarcom et al., 2015). Construction of the mutant library required generation of a linearized vector and a library of mutant N-CSP inserts. The mutant insert library was generated by two rounds of PCR using primers that carried the randomized codon “NNK” (Table S2 and Table S3) and mixing the products (Amplicons 1–9) at an equal molar ratio. The vector was generated by overlapping PCR using vector forward and reverse primers (Table S2). All PCR products were amplified using KOD DNA polymerase (EMD Millipore) and purified by gel extraction (Clontech Libraries).

The EBY100 yeast strain was purchased from ATCC. The yeast vector was generated by modification of the commercially available pCTcon2 vector (Addgene; Chao et al., 2006). The mutant N-CSP insert was cloned with N-terminal V5 and C-terminal hemagglutinin (HA) epitope tags. The *Aga2p* yeast protein gene was inserted downstream of the HA epitope tag to allow for yeast surface display of the N-CSP (plasmid pCTcon2-rsCSP-V5-HA-Aga2p). Yeast transformation was performed as described previously (Benatuil et al., 2010). In summary, 4 µg of the linearized yeast expression vector and 8 µg of the N-CSP mutant library insert were used for transformation. Transformants were plated on SDCAA plates and incubated at 30°C for 2 d. Over 10^8^ colonies were collected, resuspended in yeast extract–peptone–dextrose media with 15% glycerol, and stored at −80°C until use.

### 5D5 Fab production and purification

mAb 5D5 V_L_ and V_H_ regions were individually cloned into pcDNA3.4-TOPO expression vectors immediately upstream of human Igκ and Igγ1-C_H_1 domains, respectively. The resulting 5D5 Fab light and heavy chain vectors were cotransfected into either HEK293F or HEK293S (GnT I^−/−^) cells for transient expression and purified via KappaSelect affinity chromatography (GE Healthcare), cation exchange chromatography (MonoS; GE Healthcare), and size-exclusion chromatography (Superdex 200 Increase 10/300 GL; GE Healthcare). For binding studies, 5D5 Fab expressed in HEK293S (GnT I^−/−^) cells was digested with Endo H, followed by an additional size-exclusion chromatography step. Lastly, the 5D5Δg Fab was produced by site-directed mutagenesis of the mAb 5D5 V_H_ region using Accuprime Pfx Supermix (Thermo Fisher Scientific). 5D5Δg Fab was expressed in HEK293F cells and purified by chromatography as described above.

### IgG production and purification

For yeast display experiments, mAb 5D5 was produced in ExpiCHO cells as a mouse IgG1 with AVITag for biotinylation. The IgG was then purified using protein G affinity chromatography (HiTrap Protein G HP; GE Healthcare) and size-exclusion chromatography. Biotinylation was performed as previously described (Ekiert et al., 2011).

For production of 5D5 and 5D5Δg IgGs for non-yeast display experiments, site-directed mutagenesis was performed using In-Fusion (Takara Bio) on the pcDNA3.4-TOPO vectors encoding the 5D5 Fab heavy chain and 5D5Δg Fab heavy chain sequences to substitute two stop codons with two residues (DK), allowing for expression of the Igγ1-CH2 and Igγ1-CH3 domains. 5D5 IgG, 5D5Δg IgG, 1710 IgG (Scally et al., 2018), 1210 IgG (Imkeller et al., 2018), and IgGs elicited by Pf sporozoites with chemoprophylaxis vaccination (PfSPZ-CVac Challenge; Mordmüller et al., 2017; Murugan et al., 2018) were transiently expressed in HEK293F cells by cotransfection of paired Ig heavy and light chains and purified through protein A affinity chromatography (GE Healthcare), followed by size-exclusion chromatography.

### scFab production and purification

scFab constructs were designed by cloning paired light and heavy chains, separated by a 72-residue linker, into a pcDNA3.4-TOPO expression vector. The resulting constructs were transiently expressed in HEK293F cells and purified by KappaSelect affinity chromatography, followed by size-exclusion chromatography.

### Recombinant PfCSP production and purification

A construct of full-length PfCSP isolated from strain NF54 (UniProt accession no. P19597, residues 20–384) was designed with potential N-linked glycosylation sites mutated to glutamine (Scally et al., 2018). The resulting construct was transiently transfected in HEK293F cells and purified by HisTrap FF affinity chromatography (GE Healthcare) and size-exclusion chromatography.

A construct encoding PfCSP residues 71–104 was cloned into a pETM-22 vector. Competent BL21(DE3) *Escherichia coli* cells were transformed with the resulting plasmid and cultured to an optical density of ∼0.6–0.8. Expression of PfCSP_71–104_ was induced using 1 mM isopropyl β-D-1-thiogalactopyranoside. Approximately 4 h after induction, cells were lysed by sonication and purified through HisTrap affinity chromatography and size-exclusion chromatography.

### Yeast display epitope mapping

For each sorting round, ∼10^9^ yeast cells from the frozen stock were cultured in 250 ml of SDCAA media for 16 h at 27.5°C until an OD of 1.9 was reached. Cells were pelleted, resuspended in 35 ml of SGR-CAA (synthetic galactose, raffinose, and casamino acids) induction media, and incubated for 30 h at 18°C until an OD of 1.4 was reached. After harvesting of ∼8 ml of cell culture, the pellet was washed three times with PBS and finally resuspended in 5 ml PBS. Biotinylated 5D5 IgG was incubated with BB515-streptavidin at a molar ratio of 1:2 for 20 min. The biotinylated 5D5 IgG–streptavidin BB515 complex and anti–human HA PE antibody were added to the 5 ml of resuspended yeast cells with a final concentration of 20 nM for each stain, followed by overnight incubation at 4°C with head-to-head rotation in the dark. Next, cells were washed twice with PBS, resuspended in 5 ml of PBS, and sorted at the TSRI Flow Cytometry Core Facility. Two gates were applied for simultaneous sorting (Fig. S1 A): one where binding of 5D5 IgG was completely abrogated (PE only) and one where binding was unaffected (PE and BB515). The second round of sorting saw significant enrichment in either gate.

### Deep mutational scanning data analysis

Sequencing data were obtained in FASTQ format and parsed using the SeqIO module in BioPython (Cock et al., 2009). After the primers were trimmed, a paired-end read was then filtered and removed if the corresponding forward and reverse reads were not reverse complemented. The position of the randomized codon was then identified by the internal bar code. Each mutation was called by comparing individual paired-end reads to the WT reference sequence. Frequency of mutation *m* in sample *s* was computed as

$$Frequency_{m,s}=\frac{{\mathrm{read count}}_{\mathrm{m,s}}+1}{{\mathrm{total read count}}_{s}}.$$

Relative affinity of mutation *m* was computed as

$$\begin{array}{l} {\mathrm{Relative affinity}}_{m}=\log10\times \\ \left(\frac{{\mathrm{Frequency}}_{m,\mathrm{round 2 gate 1}}}{{\mathrm{Frequency}}_{m,\mathrm{round 2 gate 2}}}÷\frac{{\mathrm{Frequency}}_{\mathrm{WT},\mathrm{round 2 gate 1}}}{{\mathrm{Frequency}}_{\mathrm{WT},\mathrm{round 2 gate 1}}}\right). \end{array}$$

The pseudo read count of 1 in the calculation of frequency was to prevent division by zero during the calculation of relative affinity. Relative affinity of WT is 0.

Raw sequencing data have been submitted to the National Institutes of Health Short Read Archive under BioProject accession no. PRJNA578947. Custom python scripts for analyzing the deep mutational scanning data have been deposited to https://github.com/wchnicholas/CSP_Nterm_yeast_display.

### Isothermal titration calorimetry studies

Calorimetric titration experiments were performed with an Auto-iTC_200_ instrument (Malvern) at 25°C. Full-length PfCSP and PfCSP_81–98_ (GenScript) were diluted in Tris-buffered saline (TBS; 20 mM Tris, pH 8.0, and 150 mM sodium chloride) to 5–10 µM and were added to the calorimetric cell, which was titrated with 5D5 Fab, diluted to 50–100 µM in TBS, in 15 successive injections of 2.5 µl. In the reverse experimental setup, 5D5 Fab was diluted to 10 µM in TBS and added to the calorimetric cell, which was titrated with PfCSP_81–98_, diluted to 100 µM, in 15 successive injections of 2.5 µl. Experiments were performed at least three times, and the mean and SEM are reported. To investigate Pf N-CSP heparin binding, heparin sodium salt (tetrasaccharide; 9005–49-6; ApexBio) was diluted in TBS to 2,000 µM and titrated into the PfCSP_81–98_ peptide, diluted to 200 µM in TBS, in 15 successive injections of 2.5 µl at either 15°C or 25°C. The experimental data were analyzed by means of Origin 7.0 according to a 1:1 binding model.

### Size-exclusion chromatography

5D5 Fab was complexed with PfCSP_71–104_ at a 1:1 molar ratio and either coincubated with 100-fold molar excess of a high molecular heparin sodium salt (mol wt 15–19 kD; H245800; Toronto Research Chemicals) or without. The samples were loaded onto a Superdex 200 Increase 10/300 GL and compared with chromatograms of PfCSP_71–104_, 5D5 Fab, and 5D5 Fab incubated with 100-fold molar excess of the same heparin sodium salt. The peak fraction from each chromatogram was run on SDS-PAGE in nonreducing conditions.

### Crystallization and structure determination

Purified 5D5 Fab and PfCSP_81–98_ peptide were mixed in a 1:5 molar ratio. The 5D5 Fab/PfCSP_81–98_ complex was then mixed in a 1:1 ratio with 20% (wt/vol) PEG 3350, 0.2 M di-ammonium citrate. Crystals appeared after ∼20 h and were cryoprotected in 15% (vol/vol) ethylene glycol before being flash-frozen in liquid nitrogen. Data were collected at the 08ID-1 beamline at the Canadian Light Source, processed, and scaled using XDS (Kabsch, 2010). The structure was determined by molecular replacement using Phaser (McCoy et al., 2007) and an Fab model from our internal database as the search model. Refinement of the structure was performed using phenix.refine (Adams et al., 2010) and iterations of refinement using Coot (Emsley et al., 2010). The crystal structure has been deposited in the Protein Data Bank (accession no. 6UUD).

### BLI binding studies

BLI (Octet RED96; FortéBio) experiments were conducted to determine the binding kinetics of the 5D5 Fab glycoform variants to recombinant PfCSP diluted to 10 µg/ml in kinetics buffer (PBS, pH 7.4, 0.01% [wt/vol] BSA, and 0.002% [vol/vol] Tween-20) that was immobilized onto Ni-NTA biosensors (FortéBio). After a steady baseline was established, biosensors were dipped into wells containing twofold dilutions of each 5D5 Fab glycoform variant in kinetics buffer. Tips were then immersed back into kinetics buffer for measurement of the dissociation rate. Kinetics data were analyzed using FortéBio’s Data Analysis software 9.0, and curves were fitted to a 1:1 binding model. Mean kinetic constants and corresponding SD values are reported as the result of three independent experiments for each 5D5 Fab glycoform variant.

BLI experiments were also done to determine the avidity of IgGs isolated from the PfSPZ-CVac Challenge (Mordmüller et al., 2017) to full-length recombinant PfCSP and N-CSP construct PfCSP_71–104_. Unrelated Pf protein Pfs25 was used to block nonspecific binding, and 5D5 IgG was used as a positive control. PfCSP_71–104_, PfCSP, or Pfs25 was diluted to 10 µg/ml in kinetics buffer and immobilized onto Ni-NTA biosensors. Once a stable baseline was established, biosensors were dipped into wells, each containing a different IgG diluted to 500 nM in kinetics buffer. Tips were subsequently dipped back into kinetics buffer to observe any dissociation of IgG.

### Mosquito rearing, parasite infections, and sporozoite isolations

*Anopheles coluzzii* (Ngousso strain), *Anopheles gambiae* (*7b* strain; Costa et al., 2018), and *Anopheles stephensi* mosquitoes were maintained at 29°C, 70–80% humidity, 12/12–h day/night cycle. For Pf infections, *A. coluzzii* mosquitoes were fed for 15 min on a membrane feeder with NF54 gametocytes cultured with O^+^ human red blood cells (Haema) and thereafter kept in a secured BSL3 laboratory according to national regulations (Landesamt für Gesundheit und Soziales; project no. 297/13). The Pf NF54 clone used in this study originated from Prof. Sauerwein’s laboratory (Radboud University Medical Center, Nijmegen, Netherlands) and was authenticated for *Pfs47* genotype by PCR on genomic DNA. Pf asexual cultures were monthly tested for mycoplasma contamination. Unfed mosquitoes were removed shortly after infections. Blood-fed mosquitoes were offered an additional uninfected blood meal 8 d after infection, maintained at 26°C for 12 and 14/15 d, and used for the midgut and salivary gland dissections, respectively. The midgut or salivary gland sporozoites were isolated into HC-04 complete culture medium (MEM without L-glutamine [Gibco] supplemented with F-12 Nutrient Mix with L-glutamine [Gibco] in a 1:1 ratio, 15 mM Hepes, 1.5 g/L NaHCO_3_, 2.5 mM additional L-glutamine, and 10% FCS) and kept on ice until further use. The Pb sporozoites expressing the full-length Pf CSP, GFP, and luciferase (PbPfCSP) were generated as previously described (Triller et al., 2017; Flores-Garcia et al., 2019). After feeding, PbPfCSP-infected *A. gambiae 7b* strain and *A. stephensi* mosquitoes were maintained on 10% sucrose at 19–20°C, and sporozoites were harvested 18 and 20–22 d after infection for FACS and liver burden assays, respectively.

### Imaging flow cytometry of sporozoites

Concentrations of mAbs for flow cytometry studies were identified by FACS. Isolated PbPfCSP salivary gland sporozoites (Triller et al., 2017) were diluted in PBS-1% FCS to 3 × 10^6^/ml, incubated for 30 min at 4°C with mAbs (0.5 and 10.5 µg/ml), washed (16,000× *g*, 4 min, 4°C), incubated with Cy5-conjugated anti–human IgG1 (0.4 µg/ml; German Rheumatism Research Center Core Facility) for 30 min at 4°C, washed once in PBS, acquired by FACS (LSR II instrument; BD Biosciences), and analyzed with FlowJo software (version 10.0.8; Tree Star). For imaging flow cytometry analysis, Pf sporozoites were incubated with mAbs (1 µg/ml) and secondary antibody as detailed above and were additionally stained with propidium iodide (20 µg/ml; Sigma Aldrich) for 5 min at room temperature as previously described (Costa et al., 2018). After one wash in PBS, sporozoites were analyzed using the ImageStreamX Mk II instrument (Merck Millipore) with a 60× objective for 15–20 min per sample. The experiments were performed three independent times. To avoid a possible bias, the order of samples was swapped between the experimental replicates. Levels of propidium iodide staining were measured using the Intensity_MC_Ch04, whereas mAb binding was quantified by Cy5-conjugated secondary antibody signal Intensity_MC_Ch11. Single sporozoites were manually selected by brightfield images (Channel 9), and only live, propidium iodide–negative sporozoites were taken into further analysis (Fig. S3 A). The analysis was performed with IDEAS 6.2 (Merck Millipore). Raw data were exported as TXT files and represented as density plots using RStudio Version 1.1.453.

### Pf sporozoite hepatocyte traversal assay

Pf traversal assays were performed as previously described (Triller et al., 2017). Briefly, the salivary gland sporozoites were isolated from mosquito thorax and treated with mAbs (100 µg/ml) for 30 min on ice. The sporozoite preparations were seeded on human hepatocytes (HC-04; Sattabongkot et al., 2006) for 2 h at 37°C and 5% CO_2_ in the presence of dextran-rhodamine (0.5 mg/ml; Molecular Probes). mAb-untreated Pf sporozoites were used to measure the maximum traversal capacity. Cells incubated only with uninfected mosquito thoracic material were used as a background control. Cells were washed and fixed with 1% (vol/vol) formaldehyde in PBS. Dextran positivity was detected by the FACS LSR II instrument. Data analysis was performed by background subtraction and normalization to the maximum traversal capacity of mAb-untreated Pf sporozoites using FlowJo v.10.0.8.

### Parasite liver burden assay

The inhibitory activity of the mAbs was assessed in groups of five mice that were injected intravenously (tail vein) with mAbs (100 µg per mouse) and 16 h later challenged intravenously with 2,000 PbPfCSP sporozoites (Flores-Garcia et al., 2019). After 42 h, mice were injected intraperitoneally with 100 µl of D-luciferin (30 mg/ml) and anesthetized with isoflurane. Parasite liver burden was measured 5 min later using the Perkin Elmer IVIS Imaging System as luminescence generated by the transgenic sporozoites in the liver (Flores-Garcia et al., 2019).

### Passive transfer of scFabs into mosquitoes

*A. coluzzii* females (1–2 d old) were injected on ice with 100 ng (285 nl) of scFab diluted in PBS or with 285 nl PBS as an injection control. 2 d later, mosquitoes were infected with Pf NF54 following the protocol described above. Mosquito heads were carefully pulled off 14 d later, and the attached salivary glands were collected and washed with PBS. Dissected salivary glands were pooled for each group and homogenized, and the freshly isolated sporozoites were counted using a Malassez hemocytometer. The average number of sporozoites per mosquito was calculated for each group.

### Enzyme-linked immunosorbent assay

High-binding 384-well polystyrene plates (Corning) were coated with recombinantly expressed PfCSP_71–104_ comprising N-CSP at 50 ng/well overnight at 4°C. 1% BSA in PBS was used for blocking the wells at room temperature. Binding of mAbs to N-CSP was determined by incubating the coated plates with serially diluted mAb at 4.00, 1.00, 0.25, and 0.06 µg/ml concentrations. Bound mAb was detected using goat anti–human IgG–HRP (Jackson ImmunoResearch) at 1:1,000 dilution in 1% BSA in PBS and One-step ABTS substrate (Roche). Non-PfCSP reactive antibody, mGO53, was used as negative control (Wardemann et al., 2003). Area under the curve (AUC) based on diluted antibody series was calculated using GraphPad Prism 7.04. To determine the concentration of human mAbs in mouse sera collected 15 h after passive transfer, plates were coated with goat anti–human IgG (Fcγ-specific; Jackson ImmunoResearch) at 2 µg/ml. Blocking was performed with 4% BSA in PBS. Serially diluted serum samples in eight 2.5-fold steps starting with 1:200 were added. Bound mAb was detected using goat anti–human IgG–HRP at 1:1,000 dilution in 1% BSA in PBS and 1-Step ABTS substrate. A standard curve generated with human serum IgG1 (Sigma Aldrich) was used to calculate human IgG mAb concentrations.

### Statistical analysis

No samples were excluded from the analyses. Mosquitoes from the same batches were randomly allocated to the experimental groups (age range: 1–2 d). The experimenters were not blinded to the group allocation during the experiment and/or when assessing the outcome. Sample sizes were chosen according to best practices in the field and previous experience (Costa et al., 2018).

For Fig. 3 C and Fig. 4 C, MFI and SEM of the mAb-bound live sporozoites were first computed from the data. We then associated an MFI and standard error to each treatment by computing the average MFI across the three independent experiments and subsequently computing the SD as SD = [(SEM_1_^2^ + SEM_2_^2^ + SEM_3_^2^)^1/2^]/3. The null hypothesis was that the MFIs of sporozoites bound by the tested mAbs were not different. Due to the large sample sizes examined, a z-test was used to compare the MFI of the three conditions, and the obtained P values are summarized in Table 1. The combined P values from the z-test were much smaller than the total sample size, which was roughly 10^4^. P values much smaller than the inverse of the population size were therefore rounded up to 10^−4^.

**Table 1.** Summary of P values for z-test comparison of MFIs reported in Fig. 3 C and Fig. 4 C

	5D5	5D5Δg
**Fig. 3 C**		
1210	<10^−4^	<10^−4^
5D5	-	0.2
5D5Δg		-
**Fig. 4 C**	**5D5**	**5D5Δg**
1210	<10^−4^	<10^−4^
5D5	-	0.3
5D5Δg		-

Significant P values are highlighted in green.

For Fig. 3 D and Fig. 4 D, sample sizes (tot1) and proportion of mAb-bound sporozoites per experiment (pos1) for each independent experiment (*n* = 3) are summarized in Table 2.

**Table 2.** Sample sizes (tot1) and proportion of mAb-bound sporozoites per experiment (pos1) reported in Fig. 3 D and Fig. 4 D

	Exp 1		Exp 2		Exp 3	
	tot1	pos1	tot1	pos1	tot1	pos1
**Fig. 3 D**						
1710	806	0	589	1	1,121	1
1210	266	223	447	401	138	112
5D5	545	176	487	82	1096	316
5D5Δg	592	95	665	69	653	81
**Fig. 4 D**	**Exp 1**		**Exp 2**		**Exp 3**	
	**tot1**	**pos1**	**tot1**	**pos1**	**tot1**	**pos1**
1710	901	2	1,987	1	1,267	4
1210	222	219	681	675	837	831
5D5	262	67	728	229	1,456	366
5D5Δg	471	72	738	97	1,482	193

The null hypothesis was that the proportions of sporozoites bound by the tested mAbs were not different. Normality was verified, and a z-test was used to compare the fractions of mAb-bound sporozoites. We first computed the fraction *f* of mAb-bound for each mosquito tissue, experiment, and treatment as *f_i_* = pos*_i_*/tot*_i_*. The fraction *f* can be considered the probability that a sporozoite taken at random is bound by a certain mAb. The error associated to *f* is therefore given by

$$s\left(f\right)=\sqrt{f\left(1-f\right)/N},$$

where *N* is the sample size given in the column tot1. Within each experiment, we used a two-sided z-test to test the null hypothesis that the fractions *f* associated to the tested mAbs were not different, resulting in six pairwise comparisons per experiment. The resulting P values from the three independent experiments were combined using the Fisher method and are summarized in Table 3.

**Table 3.** Summary of P values for Fisher test pairwise comparisons of data reported in Fig. 3 D and Fig. 4 D

	1210	5D5	5D5Δg
**Fig. 3 D**			
1710	<10^−4^	<10^−4^	<10^−4^
1210	-	<10^−4^	<10^−4^
5D5		-	<10^−4^
**Fig. 4 D**	**1210**	**5D5**	**5D5Δg**
1710	<10^−4^	<10^−4^	<10^−4^
1210	-	<10^−4^	<10^−4^
5D5		-	<10^−4^

Significant P values are highlighted in green.

For both Fig. 3 D and Fig. 4 D, the fractions *f* organized from strong to weak are as follows: 1210, 5D5, 5D5Δg, 1710. The combined P values computed with the Fisher method are much smaller than the total sample size, which is roughly 10^4^. The reported P values are therefore the inverse of the total sample size.

Statistical analysis in Fig. 3, E and F, was performed using GraphPad Prism 8, and P values <0.05 were considered significant. Specific tests used are detailed in the corresponding figure legends.

For Fig. 4 F, the number of dissected mosquitoes and mean number of sporozoites per mosquito in each independent experiment (*n* = 6) are summarized in Table 4.

**Table 4.** Number of dissected mosquitoes and mean number of sporozoites per mosquito reported in Fig. 4 F

Dissected mosquitoes				
Exp	PBS	5D5	1710	1210
1	33	33	33	33
2	20	23	13	20
3	23	23	23	23
4	20	20	20	20
5	24	24	24	24
6	21	21	21	21
**Sporozoites per mosquito (mean)**				
**Exp**	**PBS**	**5D5**	**1710**	**1210**
1	34,285	60,612	46,394	15,510
2	22,545	15,058	10,505	8,803
3	7,733	7,304	13,743	5,333
4	6,625	5,000	4,250	3,750
5	811	1,275	579	115
6	30,095	19,428	31,047	14,666

The null hypothesis was that the average number of sporozoites for each scFab and for each experiment independently were not significantly different. To perform this test, we used the number of oocysts per mosquito from the same experiments (data available upon request). We assumed that the number of oocysts per mosquito follows a negative binomial distribution with average oocyst number *M* and shape parameter *k*:

$$\mathrm{Pr}\left(X=m|k,M\right)=\frac{\varphi \left(m+k\right)}{\varphi \left(k\right)m!}{\left(\frac{k}{M+k}\right)}^{k}{\left(\frac{M}{M+k}\right)}^{m},$$

which gives the probability that the number of oocysts, *X*, in one mosquito is equal to *m*, for *m* = 0, 1, 2, etc. We determined the two parameters *M* and *k* using a Bayesian approach with the Metropolis-Hastings algorithm and determined their maximum likelihood estimation (MLE; code available upon request). The estimates for *k* are given in Table 5.

**Table 5.** Numbers of dissected mosquitoes used for oocyst count to perform MLEs

Exp	PBS	5D5	1710	1210
1	1.15	1.07	1.37	0.97
2	0.33	0.14	0.36	0.42
3	33.3	2.44	1.76	1.37
4	0.43	0.22	0.32	0.52
5	0.47	0.38	0.56	0.36
6	0.43	0.26	0.39	0.27
**Shape parameter *k* (MLE)**				
**Exp**	**PBS**	**5D5**	**1710**	**1210**
1	21	25	15	16
2	27	18	27	28
3	11	11	11	11
4	17	22	20	19
5	19	16	16	18
6	16	20	20	17

We then assumed that the number of sporozoites was linearly proportional to the number of oocysts (Vaughan et al., 1992; Stone et al., 2013; Miura et al., 2019). This allowed us to replace *M* (as derived from oocyst distribution) in the negative binomial with the average sporozoite number as given above. We used the MLE estimate of the shape parameter *k* and simulated 10,000 independent samples of mosquitoes of the size given above. Each simulated sample is thus statistically identical to those provided by the experimental data.

Finally, for each two treatments and for each pair of simulated samples, we tested the null hypothesis that there is no difference between the treatments by random sampling while keeping sample sizes as in the experimental data. We thus created a distribution of the difference between the average number of sporozoites in the two treatments to be compared under this null hypothesis. The comparison of this distribution with the difference in sporozoite numbers as given by the experimental data produces the P values listed in Table 6. The combined P value using the Fisher method and true from false positives were discriminated using the Benjamini-Hochberg and Benjamini-Liu methods at the false discovery rate *Q* = 10^−3^.

**Table 6.** Comparison between treatments across experiments, P values, and false discovery rate analysis

Treatment		Experiment						Fisher	*Q* = 1e-3	
1	2	1	2	3	4	5	6		BH	BL
PBS	5D5	0.99	0.25	0.35	0.30	0.84	0.20	0.54	False	False
PBS	1710	0.91	0.14	0.99	0.19	0.21	0.53	0.45	False	False
PBS	1210	2e-3	0.07	0.02	0.12	0.01	0.09	8e-6	True	True
5D5	1710	0.13	0.34	0.99	0.39	0.06	0.81	0.29	False	False
5D5	1210	1e-4	0.22	0.08	0.30	0.01	0.31	1e-4	True	True
1710	1210	3e-4	0.36	1e-3	0.40	0.01	0.09	3e-6	True	True

BH, Benjamini-Hochberg; BL, Benjamini-Liu.

To check the consistency of this method, we simulated the sampled mosquito populations but skipped the shuffling across the samples.

### Animal experiment approval statement

Animal experiments were performed in accordance with Landesamt für Gesundheit und Soziales, project number H0335/17 (mosquito feeding), and the guidelines of the Institutional Animal Care and Use Committee at Johns Hopkins University, protocol number MO17H369 (liver burden assays).

### Online supplemental material

Fig. S1 presents experimental details of FACS of 5D5 IgG yeast display epitope mapping library, comparison of 5D5 Fab binding to PfCSP_81–98_ and PfCSP, and electron density observed from crystallographic studies. Fig. S2 shows investigation of heparin binding to Pf N-CSP. Fig. S3 shows the gating strategy for imaging flow cytometry quantification of mAb binding to live sporozoites, passive immunization liver burden assay, and identification of human mAbs against the Pf N-CSP from analysis of the PfSPZ-CVac samples. Table S1 presents x-ray crystallography data collection and refinement. Table S2 describes primers used to generate the mutant insert library for yeast display. Table S3 lists PCR reactions and products for mutant insert library construction.

## Supplementary Material

Table S1 presents x-ray crystallography data collection and refinement.

Table S2 describes primers used to generate the mutant insert library for yeast display.

Table S3 lists PCR reactions and products for mutant insert library construction.
