# Supplementary material for: A high-affinity antibody against the CSP N-terminal domain lacks Plasmodium falciparum inhibitory activity
Source: J Exp Med. 2020 Aug 13;217(11):e20200061. doi: 10.1084/jem.20200061 (PMC7596816; doi:10.1084/jem.20200061)
Supplement: Table S1 — presents x-ray crystallography data collection and refinement. [file JEM_20200061_TableS1.docx]

Table S1. X-ray crystallography data collection and refinement data

|  | 5D5-CSP_81–98_ |
| --- | --- |
| Wavelength (Å) | 0.97959 |
| Space group | P2_1_ |
| Cell dimensions |  |
| a, b, c (Å) | 52.6, 60.9, 72.4 |
| α, β, γ (°) | 90, 97.7, 90 |
| Resolution (Å)a | 40–1.85 (1.95–1.85) |
| No. molecules in ASU | 1 |
| No. observations | 128,170 (18,612) |
| No. unique observations | 38,412 (5,588) |
| Multiplicity | 3.3 (3.3) |
| R_merge_ (%)b | 6.5 (45.0) |
| R_pim_ (%)c | 4.2 (29.0) |
| <I/ I> | 13.7 (2.3) |
| CC_1/2_ | 99.8 (73.7) |
| Completeness (%) | 98.7 (98.7) |
| Refinement statistics |  |
| Reflections used in refinement | 38,396 |
| Reflections used for R_free_ | 1,920 |
| Non–hydrogen atoms |  |
| 5D5 Fab | 3,341 |
| PfCSP_81–98_ | 106 |
| Solvent | 420 |
| R_work_d/R_free_e | 17.8 / 22.3 |
| Rms deviations from ideality |  |
| Bond lengths (Å) | 0.005 |
| Bond angle (°) | 0.80 |
| Ramachandran plot |  |
| Favored regions (%) | 97.7 |
| Allowed regions (%) | 2.3 |
| Wilson B-value (Å^2^) | 25 |
| B-factors (Å^2^) |  |
| Average | 33 |
| 5D5 Fab | 32 |
| PfCSP_81–98_ | 50 |
| Solvent | 38 |

^a^Values in parentheses refer to the highest resolution bin.

^b^R_merge_ = hkl i | Ihkl, i − <Ihkl > | / hkl <Ihkl >.

^c^R_pim_ = hkl [1/(N − 1)]1/2 i | Ihkl, i − <Ihkl > | / hkl <Ihkl >.

^d^R_work_ = ( | |Fo | − |Fc | |) / ( | |Fo |) for all data except as indicated in footnote e.

^e^5% of data was used for the R_free_ calculation.
