# Supplementary material for: A high-affinity antibody against the CSP N-terminal domain lacks Plasmodium falciparum inhibitory activity
Source: J Exp Med. 2020 Aug 13;217(11):e20200061. doi: 10.1084/jem.20200061 (PMC7596816; doi:10.1084/jem.20200061)
Supplement: Table S2 — describes primers used to generate the mutant insert library for yeast display. [file JEM_20200061_TableS2.docx]

Table S2. Primers used to generate mutant insert library for yeast display

| Primer ID | Primer sequence |
| --- | --- |
| Condon 26 | 5′-GGCGGTAGCGGCGGAAGTNNKGGCTCTTCCAACACTCGAGTGCTGAATGAACTGAAT-3′ |
| Condon 27 | 5′-GGCGGTAGCGGCGGAAGTTACNNKTCTTCATCTAACACGCGCGTGCTGAATGAACTGAAT-3′ |
| Condon 28 | 5′-GGCGGTAGCGGCGGAAGTTACGGCNNKTCATCTAACACTCGCGTGCTGAATGAACTGAAT-3′ |
| Condon 29 | 5′-GGCGGTAGCGGCGGAAGTTACGGCTCTNNKTCGAACACTCGCGTGCTGAATGAACTGAAT-3′ |
| Condon 30 | 5′-GGCGGTAGCGGCGGAAGTTACGGGTCTTCTNNKAATACTCGCGTGCTGAATGAACTGAAT-3′ |
| Condon 31 | 5′-GGCGGTAGCGGCGGAAGTTACGGCTCTTCTTCANNKACCCGCGTGCTGAATGAACTGAAT-3′ |
| Condon 32 | 5′-GGCGGTAGCGGCGGAAGTTACGGCTCTTCGTCAAACNNKCGCGTGCTGAATGAACTGAAT-3′ |
| Condon 33 | 5′-GGCGGTAGCGGCGGAAGTTACGGCTCTTCCTCTAACACTNNKGTGCTGAATGAACTGAAT-3′ |
| Condon 34 | 5′-TCTTCTTCTAACACTCGCNNKCTCAATGAACTGAATTACGACAACGCTGGCACCCTG-3′ |
| Condon 35 | 5′-TCTTCTTCTAACACTCGCGTGNNKAATGAACTAAATTATGATAACGCTGGCACCAACCTG-3′ |
| Condon 36 | 5′-TCTTCTTCTAACACTCGCGTGCTCNNKGAACTTAATTACGATAACGCTGGCACCAACCTG-3′ |
| Condon 37 | 5′-TCTTCTTCTAACACTCGCGTGCTGAATNNKTTAAATTACGATAACGCTGGCACCAACCTG-3′ |
| Condon 38 | 5′-TCTTCTTCTAACACTCGCGTGCTCAATGAANNKAATTACGATAACGCTGGCACCAACCTG-3′ |
| Condon 39 | 5′-TCTTCTTCTAACACTCGCGTGCTTAACGAACTGNNKTACGATAACGCTGGCACCAACCTG-3′ |
| Condon 40 | 5′-TCTTCTTCTAACACTCGCGTGCTGAATGAATTGAACNNKGATAACGCTGGCACCAACCTG-3′ |
| Condon 41 | 5′-TCTTCTTCTAACACTCGCGTGTTGAATGAACTAAATTACNNKAACGCTGGCACCAACCTG-3′ |
| Condon 42 | 5′-AATGAACTGAATTACGATNNKGCAGGCACCAACCTTTATAATGAACTGGAAATGAACTAT-3′ |
| Condon 43 | 5′-AATGAACTGAATTACGATAACNNKGGCACTAACCTGTACAATGAACTGGAAATGAACTAT-3′ |
| Condon 44 | 5′-AATGAACTGAATTACGATAACGCTNNKACTAACCTGTATAATGAACTGGAAATGAACTAT-3′ |
| Condon 45 | 5′-AATGAACTGAATTACGATAACGCTGGCNNKAATCTATATAATGAACTGGAAATGAACTAT-3′ |
| Condon 46 | 5′-AATGAACTGAATTACGATAACGCGGGCACANNKCTGTATAATGAACTGGAAATGAACTAT-3′ |
| Condon 47 | 5′-AATGAACTGAATTACGATAACGCTGGCACCAACNNKTATAACGAACTGGAAATGAACTAT-3′ |
| Condon 48 | 5′-AATGAACTGAATTACGATAACGCTGGCACTAACCTCNNKAATGAACTGGAAATGAACTAT-3′ |
| Condon 49 | 5′-AATGAACTGAATTACGATAACGCTGGCACGAATCTGTATNNKGAACTGGAAATGAACTAT-3′ |
| Condon 50 | 5′-GGCACCAACCTGTATAATNNKCTGGAGATGAACTACTACGGTAAGCAGGAAAACTGGTAT-3′ |
| Condon 51 | 5′-GGCACCAACCTGTATAATGAANNKGAGATGAACTATTATGGTAAGCAGGAAAACTGGTAT-3′ |
| Condon 52 | 5′-GGCACCAACCTGTATAATGAACTGNNKATGAACTACTACGGGAAGCAGGAAAACTGGTAT-3′ |
| Condon 53 | 5′-GGCACCAACCTGTATAATGAACTCGAANNKAATTATTACGGTAAGCAGGAAAACTGGTAT-3′ |
| Condon 54 | 5′-GGCACCAACCTGTATAATGAACTTGAAATGNNKTATTATGGTAAGCAGGAAAACTGGTAT-3′ |
| Condon 55 | 5′-GGCACCAACCTGTATAATGAACTAGAAATGAACNNKTACGGCAAGCAGGAAAACTGGTAT-3′ |
| Condon 56 | 5′-GGCACCAACCTGTATAATGAATTGGAAATGAACTATNNKGGCAAGCAGGAAAACTGGTAT-3′ |
| Condon 57 | 5′-GGCACCAACCTGTATAATGAATTGGAAATGAACTATTACNNKAAGCAGGAAAACTGGTAT-3′ |
| Condon 58 | 5′-GAAATGAACTATTACGGTNNKCAGGAAAACTGGTACAGCCTTAAAAAGAACAGCCGCAGC-3′ |
| Condon 59 | 5′-GAAATGAACTATTACGGTAAGNNKGAGAACTGGTATAGCTTGAAAAAGAACAGCCGCAGC-3′ |
| Condon 60 | 5′-GAAATGAACTATTACGGTAAGCAGNNKAACTGGTACAGCCTGAAAAAGAACAGCCGCAGC-3′ |
| Condon 61 | 5′-GAAATGAACTATTACGGTAAGCAGGAANNKTGGTATAGTTTGAAAAAGAACAGCCGCAGC-3′ |
| Condon 62 | 5′-GAAATGAACTATTACGGTAAGCAGGAAAACNNKTACAGCCTCAAAAAGAACAGCCGCAGC-3′ |
| Condon 63 | 5′-GAAATGAACTATTACGGTAAGCAGGAAAACTGGNNKAGCCTAAAAAAGAACAGCCGCAGC-3′ |
| Condon 64 | 5′-GAAATGAACTATTACGGTAAGCAAGAAAACTGGTATNNKCTTAAAAAGAACAGCCGCAGC-3′ |
| Condon 65 | 5′-GAAATGAACTATTACGGTAAGCAGGAAAATTGGTATAGTNNKAAAAAGAACAGCCGCAGC-3′ |
| Condon 66 | 5′-GAAAACTGGTATAGCCTGNNKAAGAACAGCCGCAGTCTCGGTGAAAACGACGACGGTAAC-3′ |
| Condon 67 | 5′-GAAAACTGGTATAGCCTGAAANNKAATAGTCGCAGCCTGGGTGAAAACGACGACGGTAAC-3′ |
| Condon 68 | 5′-GAAAACTGGTATAGCCTGAAAAAGNNKAGCCGGAGCCTTGGTGAAAACGACGACGGTAAC-3′ |
| Condon 69 | 5′-GAAAACTGGTATAGCCTGAAAAAGAACNNKAGGAGCCTGGGTGAAAACGACGACGGTAAC-3′ |
| Condon 70 | 5′-GAAAACTGGTATAGCCTGAAAAAGAACAGCNNKAGCCTCGGGGAAAACGACGACGGTAAC-3′ |
| Condon 71 | 5′-GAAAACTGGTATAGCCTGAAAAAGAACAGCCGCNNKCTCGGCGAAAACGACGACGGTAAC-3′ |
| Condon 72 | 5′-GAAAACTGGTATAGCCTGAAAAAGAACAGCCGCAGCNNKGGAGAAAACGACGACGGTAAC-3′ |
| Condon 73 | 5′-GAAAACTGGTATAGCCTGAAAAAGAACAGCCGTAGTCTGNNKGAAAACGACGACGGTAAC-3′ |
| Condon 74 | 5′-AACAGCCGCAGCCTGGGTNNKAACGACGACGGCAATAACGAGGACAATGAAAAACTGCGC-3′ |
| Condon 75 | 5′-AACAGCCGCAGCCTGGGTGAANNKGATGACGGAAACAACGAGGACAATGAAAAACTGCGC-3′ |
| Condon 76 | 5′-AACAGCCGCAGCCTGGGTGAAAACNNKGATGGCAACAACGAGGACAATGAAAAACTGCGC-3′ |
| Condon 77 | 5′-AACAGCCGCAGCCTGGGTGAAAACGACNNKGGTAATAACGAGGACAATGAAAAACTGCGC-3′ |
| Condon 78 | 5′-AACAGCCGCAGCCTGGGTGAAAATGACGACGACNNKAACAACGAGGACAATGAAAAACTGCGC-3′ |
| Condon 79 | 5′-AACAGCCGCAGCCTGGGTGAAAATGACGACGGTNNKAACGAGGACAATGAAAAACTGCGC-3′ |
| Condon 80 | 5′-AACAGCCGCAGCCTGGGTGAAAACGACGACGGCAACNNKGAAGACAATGAAAAACTGCGC-3′ |
| Condon 81 | 5′-AACAGCCGCAGCCTGGGTGAAAATGACGACGGCAACAACNNKGACAATGAAAAACTGCGC-3′ |
| Condon 82 | 5′-GACGACGGTAACAACGAGNNKAATGAAAAGCTGCGTAAGCCTAAACACAAAAAGCTGAAA-3′ |
| Condon 83 | 5′-GACGACGGTAACAACGAGGACNNKGAAAAATTGCGCAAACCTAAACACAAAAAGCTGAAA-3′ |
| Condon 84 | 5′-GACGACGGTAACAACGAGGACAATNNKAAATTGCGGAAGCCTAAACACAAAAAGCTGAAA-3′ |
| Condon 85 | 5′-GACGACGGTAACAACGAGGACAACGAANNKCTGCGCAAGCCAAAACACAAAAAGCTGAAA-3′ |
| Condon 86 | 5′-GACGACGGTAACAACGAGGACAATGAGAAANNKCGAAAGCCTAAACACAAAAAGCTGAAA-3′ |
| Condon 87 | 5′-GACGACGGTAACAACGAGGACAATGAAAAGCTGNNKAAACCTAAACACAAAAAGCTGAAA-3′ |
| Condon 88 | 5′-GACGACGGTAACAACGAGGACAACGAAAAACTGCGCNNKCCTAAACACAAAAAGCTGAAA-3′ |
| Condon 89 | 5′-GACGACGGTAACAACGAGGACAATGAAAAACTACGCAAGNNKAAACACAAAAAGCTGAAA-3′ |
| Condon 90 | 5′-GAAAAACTGCGCAAGCCTNNKCACAAGAAGCTGAAACAGCCTGCGGACGGTAATCCGGAT-3′ |
| Condon 91 | 5′-GAAAAACTGCGCAAGCCTAAANNKAAAAAACTCAAACAGCCGGCGGACGGTAATCCGGAT-3′ |
| Condon 92 | 5′-GAAAAACTGCGCAAGCCTAAACACNNKAAACTGAAACAGCCGGCGGACGGTAATCCGGAT-3′ |
| Condon 93 | 5′-GAAAAACTGCGCAAGCCTAAACACAAANNKTTGAAGCAGCCGGCGGACGGTAATCCGGAT-3′ |
| Condon 94 | 5′-GAAAAACTGCGCAAGCCTAAACACAAAAAGNNKAAACAGCCGGCGGACGGTAATCCGGAT-3′ |
| Condon 95 | 5′-GAAAAACTGCGCAAGCCTAAACACAAGAAGCTGNNKCAACCGGCGGACGGTAATCCGGAT-3′ |
| Condon 96 | 5′-GAAAAACTGCGCAAGCCTAAAAAGCTTAAGNNKCCGGCGGACGGTAATCCGGAT-3′ |
| Condon 97 | 5′-GAAAAACTGCGCAAGCCTAAACATAAGAAGCTGAAACAGNNKGCGGACGGTAATCCGGAT-3′ |
| Fragment 1-R | 5′-ACTTCCGCCGCTACCGCCTGTAGAATCTAG-3′ |
| Fragment 2-R | 5′-GCGAGTGTTAGAAGAAGAGCCGTAACTTCC-3′ |
| Fragment 3-R | 5′-ATCGTAATTCAGTTCATTCAGCACGCGAGT-3′ |
| Fragment 4-R | 5′-ATTATACAGGTTGGTGCCAGCGTTATCGTA-3′ |
| Fragment 5-R | 5′-ACCGTAATAGTTCATTTCCAGTTCATTATA-3′ |
| Fragment 6-R | 5′-CAGGCTATACCAGTTTTCCTGCTTACCGTA-3′ |
| Fragment 7-R | 5′-ACCCAGGCTGCGGCTGTTCTTTTTCAGGCT-3′ |
| Fragment 8-R | 5′-CTCGTTGTTACCGTCGTCGTTTTCACCCAG-3′ |
| Fragment 9-R | 5′-AGGCTTGCGCAGTTTTTCATTGTCCTCGTT-3′ |
| Universal-F | 5′-TCTGTTATTGCTTCAGTTTTAGCAGCGGCC-3′ |
| Universal-R | 5′-CCGTTACCGCACGTGACAGAGCACGGGCTC-3′ |
| Vector-F | 5′-AATAGCCTGTCCACCGAATGGAGCCCGTGCTCTGTCACGTGCGGTAACGG-3′ |
| Vector-R | 5′-TGGCTTTCCGCCGGCCGGCTGGGCCGCTGCTAAAACTGAAGCAATAACAGA-3′ |
