# Supplementary material for: A high-affinity antibody against the CSP N-terminal domain lacks Plasmodium falciparum inhibitory activity
Source: J Exp Med. 2020 Aug 13;217(11):e20200061. doi: 10.1084/jem.20200061 (PMC7596816; doi:10.1084/jem.20200061)
Supplement: Table S3 — lists PCR reactions and products for mutant insert library construction. [file JEM_20200061_TableS3.docx]

Table S3. PCR reactions and products for mutant insert library construction

| Reactiom ID | Forward primer | Reverse primer | Template | Product name |
| --- | --- | --- | --- | --- |
| Round 1 reaction #1 | Equal molar ratio of Codon 26 to Codon 33 | Universal-R | pCTcon2-rsCSP-V5-HA-Aga2p | Fragment 1-1 |
| Round 1 reaction #2 | Equal molar ratio of Codon 34 to Codon 41 | Universal-R | pCTcon2-rsCSP-V5-HA-Aga2p | Fragment 2-1 |
| Round 1 reaction #3 | Equal molar ratio of Codon 42 to Codon 49 | Universal-R | pCTcon2-rsCSP-V5-HA-Aga2p | Fragment 3-1 |
| Round 1 reaction #4 | Equal molar ratio of Codon 50 to Codon 57 | Universal-R | pCTcon2-rsCSP-V5-HA-Aga2p | Fragment 4-1 |
| Round 1 reaction #5 | Equal molar ratio of Codon 58 to Codon 65 | Universal-R | pCTcon2-rsCSP-V5-HA-Aga2p | Fragment 5-1 |
| Round 1 reaction #6 | Equal molar ratio of Codon 66 to Codon 73 | Universal-R | pCTcon2-rsCSP-V5-HA-Aga2p | Fragment 6-1 |
| Round 1 reaction #7 | Equal molar ratio of Codon 74 to Codon 81 | Universal-R | pCTcon2-rsCSP-V5-HA-Aga2p | Fragment 7-1 |
| Round 1 reaction #8 | Equal molar ratio of Codon 82 to Codon 89 | Universal-R | pCTcon2-rsCSP-V5-HA-Aga2p | Fragment 8-1 |
| Round 1 reaction #9 | Equal molar ratio of Codon 90 to Codon 97 | Universal-R | pCTcon2-rsCSP-V5-HA-Aga2p | Fragment 9-1 |
| Round 1 reaction #10 | Universal-F | Fragment 1-R | pCTcon2-rsCSP-V5-HA-Aga2p | Fragment 1-2 |
| Round 1 reaction #11 | Universal-F | Fragment 2-R | pCTcon2-rsCSP-V5-HA-Aga2p | Fragment 2-2 |
| Round 1 reaction #12 | Universal-F | Fragment 3-R | pCTcon2-rsCSP-V5-HA-Aga2p | Fragment 3-2 |
| Round 1 reaction #13 | Universal-F |  | pCTcon2-rsCSP-V5-HA-Aga2p | Fragment 4-2 |
| Round 1 reaction #14 | Universal-F | Fragment 4-R | pCTcon2-rsCSP-V5-HA-Aga2p | Fragment 5-2 |
| Round 1 reaction #15 | Universal-F | Fragment 5-R | pCTcon2-rsCSP-V5-HA-Aga2p | Fragment 6-2 |
| Round 1 reaction #16 | Universal-F | Fragment 6-R | pCTcon2-rsCSP-V5-HA-Aga2p | Fragment 7-2 |
| Round 1 reaction #17 | Universal-F | Fragment 7-R | pCTcon2-rsCSP-V5-HA-Aga2p | Fragment 8-2 |
| Round 1 reaction #18 | Universal-F | Fragment 8-R | pCTcon2-rsCSP-V5-HA-Aga2p | Fragment 9-2 |
| Round 2 reaction #1 | Universal-F | Fragment 9-R | Equal molar ratio of Fragment 1-2 and Fragment 1-2 | Amplicon 1 |
| Round 2 reaction #2 | Universal-F | Universal-R | Equal molar ratio of Fragment 2-2 and Fragment 2-2 | Amplicon 2 |
| Round 2 reaction #3 | Universal-F | Universal-R | Equal molar ratio of Fragment 3-2 and Fragment 3-2 | Amplicon 3 |
| Round 2 reaction #4 | Universal-F | Universal-R | Equal molar ratio of Fragment 4-2 and Fragment 4-2 | Amplicon 4 |
| Round 2 reaction #5 | Universal-F | Universal-R | Equal molar ratio of Fragment 5-2 and Fragment 5-2 | Amplicon 5 |
| Round 2 reaction #6 | Universal-F | Universal-R | Equal molar ratio of Fragment 6-2 and Fragment 6-2 | Amplicon 6 |
| Round 2 reaction #7 | Universal-F | Universal-R | Equal molar ratio of Fragment 7-2 and Fragment 7-2 | Amplicon 7 |
| Round 2 reaction #8 | Universal-F | Universal-R | Equal molar ratio of Fragment 8-2 and Fragment 8-2 | Amplicon 8 |
| Round 2 reaction #9 | Universal-F | Universal-R | Equal molar ratio of Fragment 9-2 and Fragment 9-2 | Amplicon 9 |
